# Tricaine methanesulfonate and eugenol during semen collection do not affect fertilization and hatching rates on *Rhamdia quelen*

**DOI:** 10.1590/1984-3143-AR2024-0107

**Published:** 2026-03-30

**Authors:** Nathalia dos Santos Teixeira, Lis Santos Marques, Rômulo Batista Rodrigues, Thales de Souza França, Jhony Lisbôa Benato, Maritza Pérez Atehortúa, Darlan Gusso, Itamar Cossina Gomes, Thaiza Rodrigues de Freitas, Henrique Mautone Gomes, Helen Tais Rosa-Silva, Alexandre Kleber Silveira, José Claudio Fonseca Moreira, Danilo Pedro Streit

**Affiliations:** 1 Grupo de Pesquisa Aquam, Programa de Pós-graduação em em Ciências Veterinárias, Universidade Federal do Rio Grande do Sul – UFRGS, Porto Alegre, Brasil; 2 Departamento de Zootecnia e Ciências Biológicas, Universidade Federal de Santa Maria – UFSM, Palmeira das Missões, Brasil; 3 Universitat Politècnica de València, Instituto de Ciencia y Tecnología Animal, Valencia, Valencia, Spain.; 4 Programa de Pós-graduação em Zootecnia, Universidade Federal do Rio Grande do Sul – UFRGS, Porto Alegre, Brasil; 5 Departamento de Ciencias Agrarias y Acuícolas, Facultad de Recursos Naturales, Universidad Católica de Temuco, Temuco, Chile; 6 Laboratório de Neuroquímica e Psicofarmacologia, Programa de Pós-graduação em Biologia Celular e Molecular, Escola de Ciências, Pontifícia Universidade Católica do Rio Grande do Sul, Porto Alegre, Brasil; 7 Aquatic Germplasm and Genetic Resources Center, Louisiana State University Agricultural Center, Baton Rouge, USA; 8 Centro de Estudos em Estresse Oxidativo, Programa de Pós-graduação em Ciências Biológicas: Bioquímica, Instituto de Ciências Básicas da Saúde, Universidade Federal do Rio Grande do Sul – UFRGS, Porto Alegre, Brasil

**Keywords:** DNA damage, fish anesthesia, fish welfare, oxidative stress, reproductive management

## Abstract

The reproduction of some fish species in captivity is only possible by imposing artificial reproductive procedures, and the manipulation of fish for these purposes is a stressor. Thus, anesthesia can reduce stress during handling. However, it is necessary to investigate the possible side effects on breeding and the general health of the animal. Therefore, we aimed to investigate the impact of tricaine methanesulfonate (MS-222) and eugenol (EUG) at concentrations of 300 mg L^-1^ and 40 mg L^-1^, respectively, on several stress- and reproductive-related parameters in *Rhamdia quelen* anesthetized before semen collection. After hormonal induction, 24 sexually mature males (534.4 ± 259 g) were randomly assigned to treatment groups and semen and blood samples were collected. Anesthesia recovery and induction time were extended in fish anesthetized with EUG; however, plasma cortisol concentrations did not differ among the treatment groups. EUG caused higher DNA fragmentation in blood cells than in the control group (without anesthesia). EUG increased the monocyte count compared to the other experimental groups. MS-222 showed a lower sulfhydryl group (SH) quantitation than the other experimental groups. The anesthetics used before semen collection in this study had no adverse effects on the fertilization or hatching capacity of anesthetized *R. quelen*. MS-222 (300 mg L^-1^) and EUG (40 mg L^-1^), despite being related to hematological and semen changes, did not negatively affect the reproductive capacity of *R. quelen* males. Thus, anesthesia is recommended for *R. quelen* during semen collection, considering reproductive parameters and animal welfare.

## Introduction

Concerns about animal welfare in production systems and scientific experimentation have gained prominence in recent years, particularly in fish research (Campbell et al., 2019; Gouveia and Hurst, 2019; Bojarski et al., 2025). Fish, as sentient aquatic organisms, are increasingly affected by anthropogenic activities, especially those related to aquaculture practices (Macaulay et al., 2020; Aravena-Canales et al., 2025). Evidence shows that fish possess the neurobiological mechanisms for nociception and pain perception (Sneddon, 2019; Ohnesorge et al., 2021), reinforcing the need for appropriate anesthesia during routine aquaculture procedures such as spawning, handling, and sampling (Fish et al., 2008; Ross and Ross, 2008; Azizi et al., 2025). Also, providing proper anesthesia is therefore an important requirement for many animal experiments to ensure animal welfare, wellbeing and data reliability.

Anesthesia induces a reversible, generalized loss of sensation accompanied by a sleep-like state resulting from central nervous system depression. Among the anesthetics used in fish, MS-222 (tricaine methanesulfonate; ethyl 3-aminobenzoate methanesulfonate) is the most widely used agent for anesthesia, sedation, and euthanasia via immersion baths (Popovic et al., 2012; Collymore et al., 2016; Wojan et al., 2019; Lavalle et al., 2025). Although some studies have reported avoidance behavior in certain fish species during MS-222 exposure (Popovic et al., 2012; Wong et al., 2014; Ferreira et al., 2022), this compound is considered a safe and fast-acting anesthetic on the central nervous system, as demonstrated in zebrafish larvae (Ohnesorge et al., 2024). Notably, MS-222 is the only anesthetic approved by the U.S. Food and Drug Administration (FDA) for use in fish intended for human consumption. The recommended concentration for anesthesia induction ranges from 50 to 400 mg L^−1^, depending on the species (Sneddon, 2012).

Plant oils, such as eugenol (4-allyl-2-methoxyphenol) extracted from the *Eugenia caryophyllata* plant (Kamatou et al., 2012; Silva et al., 2013; Hoseini et al., 2018) have been used as natural anesthetics in fishes (Salbego et al., 2017; Tago et al., 2017; Oliveira et al., 2019a; Oliveira et al., 2019b; Dong et al., 2020; Nascimento et al., 2025).These substances are considered safe for humans and are classified as GRAS (Generally Recognized as Safe) by the world's leading chemical regulatory agency, the US Food and Drug Administration (FDA). Although it is widely used as anesthetic in fish research, it is known that eugenol can modulate plasma cortisol levels (Corso et al., 2019), and it could be potentially toxic to the fish brain (Barbas et al., 2021).

The South American catfish *Rhamdia quelen* (Siluriformes, Heptapteridae) is a native freshwater species that lives in lakes and rivers and prefers calm water environments. This species has been widely studied in terms of various aspects related to reproduction (Goes et al., 2017; Hilbig et al., 2019; Corso et al., 2019; Pérez-Atehortúa et al., 2022; França et al., 2023; Coimbra et al., 2025) and anesthetic procedures (Gressler et al., 2014, 2015; Fortes et al., 2024).

Research on the anesthetics used in breeding fish and the effects of these drugs on gamete viability is limited. A study carried out on rainbow trout, *Oncorhynchus mykiss* (Wagner et al., 2002) showed that anesthesia with MS-222, AQUI-L, and carbon dioxide caused a decrease in the duration of spermatozoa motility. Other studies have also reported a negative impact on semen from fish anesthetized with MS-222, including Salvelinus *fontinalis* (Allison, 1961), *Danio rerio* (Zanin et al., 2021); *Rhamdia quelen* (Teixeira et al., 2021), and rainbow trout (Dietrich et al., 2006). Therefore, it is necessary to elucidate the effects of these anesthetics on male gametes. Therefore, the present study aimed to analyze the impact of MS-222 and eugenol on parameters of stress and reproductive efficiency of *R. quelen* subjected to anesthesia during semen collection.

## Methods

### Ethics statement

All experimental procedures imposed on animals were approved by the Animal Use Ethics Committee of the Federal University of Rio Grande do Sul (CEUA - UFRGS) (project 35840), and all procedures used were consistent with the established guidelines of the National Council for the Control of Animal Experimentation (CONCEA).

### Fish maintenance and experimental conditions

Two-year-old South American silver catfish males (n = 24; 534,4 ± 259 g) and females (n = 6; 857 ± 28g) were acclimated in four plastic tanks (500 L) with a black background in a recirculating water system with temperature (26°C) and photoperiod control (14h light/10h dark) for four weeks before the experiment. The experimental period was 40 days and was conducted during the summer in southern Brazil. Fish were fed twice daily (8 a.m. and 4 p.m.) with a commercial diet (32% crude protein, Acqua Fish, Supra^®^, Alisul, Brazil) until apparent satiety. Animal health (skin and fin integrity) and behavior (swimming and feeding activity) were monitored daily during feeding time. The experimental parameters for water are listed in Table 1.

**Table 1:** Parameters of the water vessel during the experiment. Hardness, dissolved oxygen, nitrite, and ammonia were measured through kits for water analyses (LabconTest, USA). The temperature was measured using a digital thermometer (AKSO, Brazil) and pH through a pHmeter (K39-2014B, Benchtop pH meter, Kasvi, China).

**Water parameter**	**Control**	**EUG**	**MS-222**
Hardness (mg L^-1^ CaCO_3_)	50.7 ± 6.7	48.9 ± 8.2	99.5± 27.6
Dissolved oxygen (mg L^-1^)	5.5 ± 0.5	5.5 ± 0.5	5.5 ± 0.5
Nitrite (mg L^-1^)	0	0	0
Ammonia (mg L^-1^)	0	0	0
pH	7.2 ± 0.1	7.4 ± 0.1	7 ± 0.1
Temperature (ºC)	25.57 ± 0.3	25.2 ± 0.4	24.9 ± 0.3

Experiments on fish were conducted using procedures consistent with the ARRIVE guidelines and the National Institutes of Health Guide for the Care and Use of Laboratory Animals (NIH Publication No. 8023, revised 1978). In all manipulations (collection of semen and blood), the fish were covered with damp towels and eyes and handled with wool gloves to protect the keeper and the animal.

All efforts were made to minimize animal stress (i.e., handling care, quiet environments, and maintenance of water quality). After anesthesia, the fish were maintained in a 500 L anesthetic-free water tank to recover, and their health and behavior were observed for 96 h. No behavioral or clinical changes were observed in the fish, and no mortality resulted from the experimental procedures.

### Experimental design

**Figure 1:**
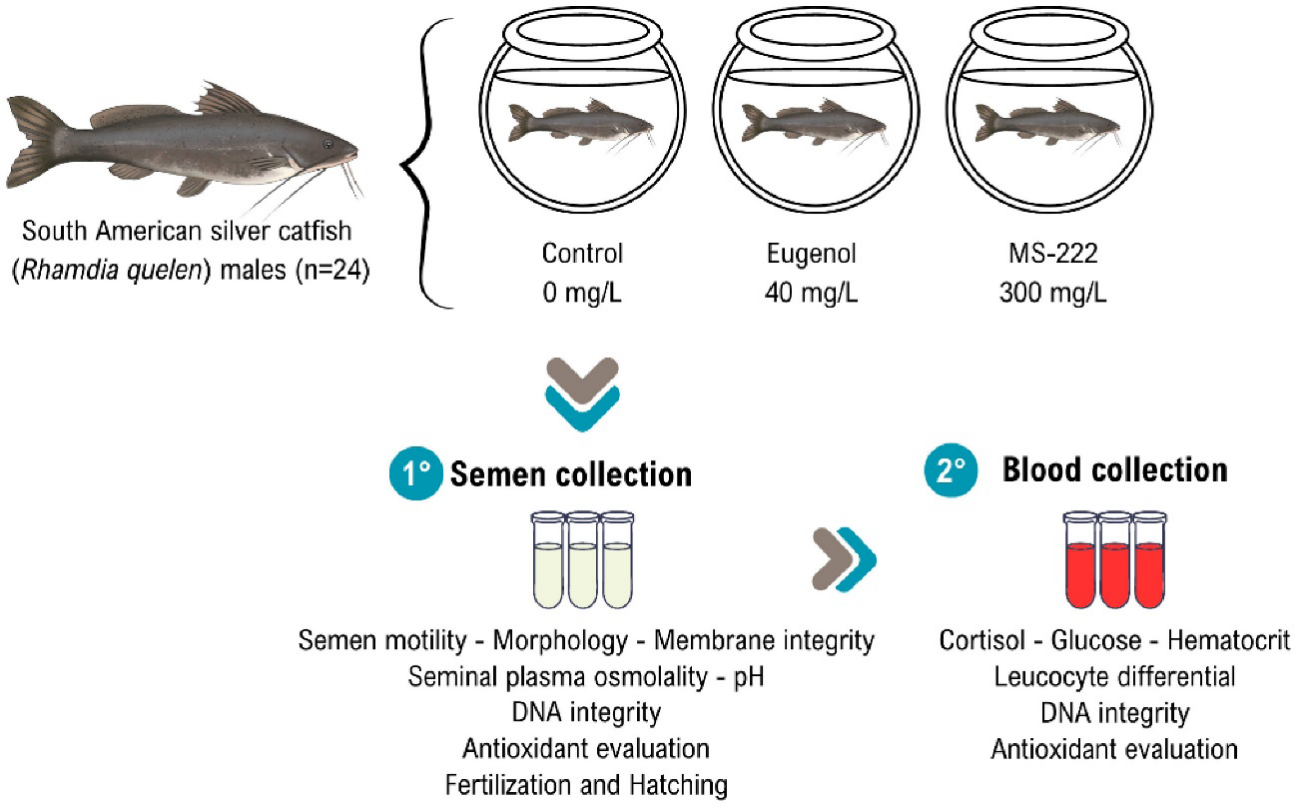
Experimental design to compare two different anesthetics (MS-222 and eugenol) applied to semen collection in catfish *(Rhamdia quelen*). Firstly, the males were exposed individually to an anesthetic bath containing MS-222 or eugenol or a bath without anesthetic. After achieving deep anesthesia, the semen was collected and analyzed. After the semen collection, it was realized the blood puncture and subsequent validations.

The experimental design is illustrated in Figure 1. The experiment was performed using a completely randomized design, with each male considered a repetition. Three treatments were compared: MS-222 (Sigma-Aldrich, USA, CAS Number: 886-86-2) at a concentration of 300 mg L^-1^ (Gressler et al., 2012; Teixeira et al., 2021), Eugenol (Biodinâmica, Brazil) at a concentration of 40 mg L^-1^ (Corso et al., 2019), and a control treatment where the animals were not anesthetized. Each treatment group consisted of eight males (each animal was considered a repetition). The control group specimens were placed in water without anesthetic to mimic the average duration of anesthesia that occurred in the treated specimens.

### Anesthetic baths

Anesthetic bath treatments were performed individually for each fish prior to semen collection. MS-222 and eugenol were prepared as previously described (Ross and Ross, 2008). EUG was first diluted in ethanol 70% (1:9). Subsequently, each male was carefully removed from the maintenance tank and placed inside a vessel containing MS-222, eugenol, or a water without anesthetic. Anesthesia induction based on fish anesthesia stage classifications was considered to achieve deep anesthesia (IV) (Ross and Ross, 2008). Under Stage IV anesthesia, the animal loses muscle tone and balance with slow but regular opercular movements. The anesthesia induction solutions were replaced with new solutions for each animal to ensure that all specimens were treated with the intended concentrations of the anesthetic. The anesthesia induction time was monitored using a digital stopwatch, and the time (s) each fish was immersed in the solution until there was a total loss of movement and a marked decrease in opercular movement. For recovery from the anesthetic procedure, after semen and blood collection, the fish were returned to the maintenance tank, where the water was free from anesthetic compounds. As soon as the fish were placed in the system, the time until equilibrium was recovered was recorded using a digital stopwatch. Furthermore, the handling time (time needed to collect semen and blood) was recorded between reaching anesthesia and returning to the maintenance tank.

### Hormonal induction and semen collection

Hormonal induction of *R. quelen* males was performed by intramuscular application of carp pituitary extract (CPE) at a concentration of 3 mg/kg (pituitary/fish weight) using an insulin syringe (1 mL) and a 13 × 0.45 mm needle. No type of anesthesia was used during the hormonal induction procedure. The temperature was maintained at 26.0 ± 0.5°C in a recirculating system with temperature control. After a thermal accumulation of about 240 hours-degree (Sanches et al., 2011) (9,23 h at 26 ºC) (the time the fish will take to spawn depending on the temperature of the system in which the specimens are housed), the fish were placed in a recipient with 5 L of water containing the different anesthetic concentrations according with the treatment group. For semen collection, each male was slightly tilted with the head up, and an anteroposterior massage was applied to the abdominal region until the semen flowed. The first drop was discarded to avoid contamination with water, urine, or blood. The semen was collected in 15 mL graduated tubes (Falcon® Conical Centrifuge tube) until the release was completed. Samples were collected from all fish (*n* = 24; eight per treatment). After the collection, the semen was stored in a refrigerator (6 °C ±1 °C) for 30 min until the start of the evaluation.

The hormonal induction protocol for sexually mature female of *R. quelen* was performed with intramuscular application of CPE. The first application at a concentration of 0.5 mg/kg (CPE/fish weight) was carried out 12 h before the second application, which was carried out at a concentration of 5 mg/kg (CPE/fish weight). An insulin syringe (1 mL) and a 13 × 0.45 mm needle were used for both applications. The temperature was maintained at 26.0 ± 0.5°C in a recirculating system with temperature control. After a thermal accumulation of 240 degree-hours from the second CPE application (10 h at a temperature of 24°C), the females were removed individually from the tanks. Oocyte collection was performed by applying an anteroposterior massage to the abdominal region and collecting the oocytes in a 1000 mL Becker cup. During oocyte collection, contamination with feces, blood, or urine was avoided by cleaning the urogenital region with clean tissues.

### Semen analyses

#### Seminal plasma osmolality and pH

A semen aliquot (2 mL) from each male was collected in a plastic tube and centrifuged (Benchtop Centrifuge; Edutec, Astral Científica, Brazil) at 3000 × g for 10 min. The collected seminal plasma (supernatant) was frozen (-20° C) and subsequently evaluated for pH using a pH meter (K39-2014B; Benchtop pH meter, Kasvi, China). The osmolality was tested using an automatic freezing-point osmometer (5004 Micro-Osmette; Precision Systems, USA).

#### Spermatozoa motility, morphology, and membrane integrity

Immediately after collection, each semen sample was subjectively evaluated under a light microscope (Nikon E200, Japan) at 400 × magnification to check for possible previous activation of spermatozoa by contaminants or water, motility rate (0–100%), and duration of motility (s) (Carolsfeld et al., 2003). A semen sample from each male (1 μL) was activated using distilled water (5000 μL) at a ratio of 1:5000 (semen: distilled water) (Neumann et al., 2019).

**Figure 2:**
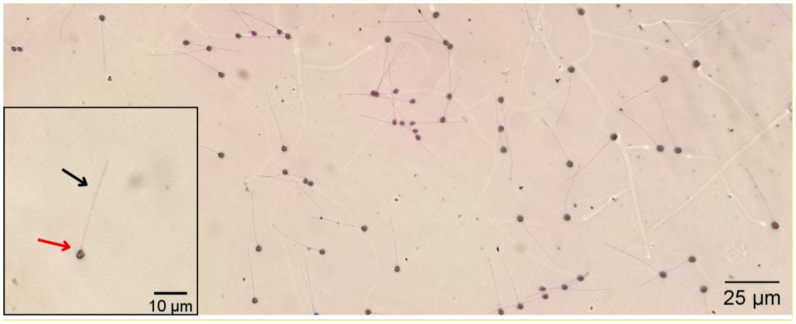
Micrograph of a *Rhamdia quelen* spermatozoa stained with Bengal Rose. The black arrow indicates the tail, and the red arrow indicates the head.

To evaluate spermatozoa morphology, semen samples were fixed in 10% buffered formalin solution at 1:1000 dilution. From the fixed semen, 100 µL were stained with 10 µL Bengal Rose dye (4%) (Merck, Germany) for cellular morphology evaluation (Streit-Junior et al., 2004) (Figure 2). Smears of 20 μL of stained semen were evaluated under an optical microscope at 1000 × magnification (Nikon E200, Tokyo, Japan). Spermatozoa (n = 300) were evaluated from each sample (n = 24 males), and the number of normal and abnormal cells was expressed as a percentage. Spermatozoa morphological changes were classified into head abnormalities (loose head, degeneration, macrocephaly, and microcephaly) and flagella abnormalities (broken tail, strongly coiled tail, distally curled tail, short flagellum, folded tail, and proximal and distal cytoplasmic gouts) (Miliorini et al., 2011).

The percentage of cells with intact membranes was evaluated using the eosin–nigrosin dye protocol adapted from Blom (1950), using the dyes Eosin Y (3%; w:v) and nigrosin (5%; w:v), both diluted in 3% (w/v) sodium citrate. A semen aliquot of 20 μL was dyed with 10 μL of each dye. Later, 10 μL was used to make the slide-through smear. After completion and air-drying, the slides were photographed using a smartphone (iPhone XR, Apple, Los Altos, USA) coupled to an optical microscope (Nikon E200, Japan) at 400X magnification. Photographs were transferred to a computer and analyzed using a plug-in Cell Counter (ImageJ). The percentage of spermatozoa with intact membranes was quantified as 300 cells per male, considering spermatozoa with an unstained head intact.

#### Fertilization and hatching

**Figure 3:**
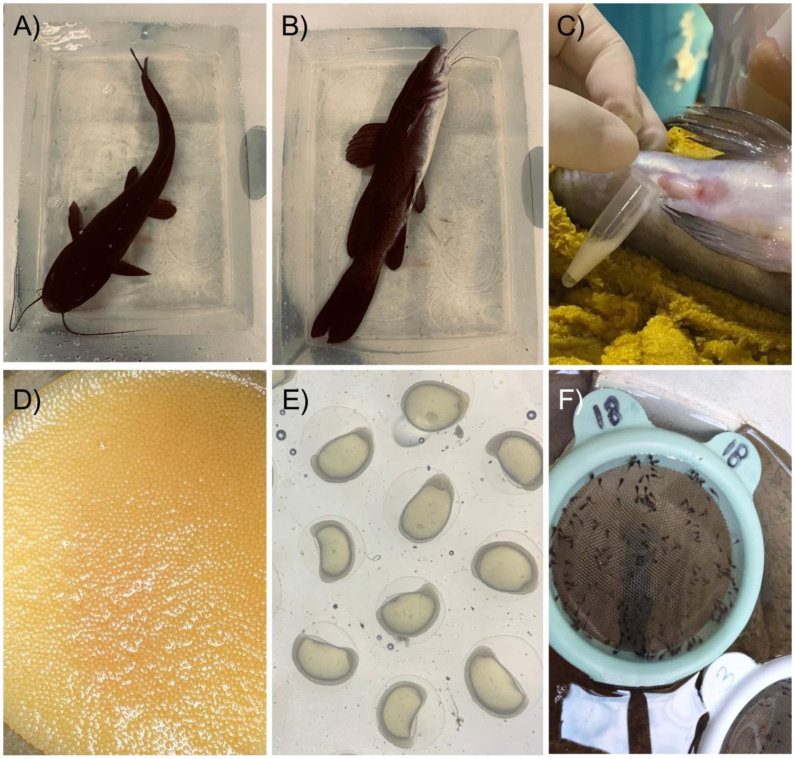
Reproductive stages of *Rhamdia quelen.* A) exposure of the fish to anesthetic; B) attainment of the anesthetic plane; C) semen collection; D) oocytes obtained from females; E) embryo development; F) hatched larvae after incubation in mesh sieves.

Oocytes obtained after hormone induction with CPE were used in equal quantities (approximately 800 oocytes) and mixed with the volume of semen indicated for each treatment (control, MS-222, or EUG). The inseminating dose (Bombardelli et al., 2006) was calculated from the semen concentration, allowing 90,000 spermatozoa per oocyte. The sperm concentration was analyzed with a Neubauer chamber, with previous fixation of the sperm in saline-buffered formaldehyde (4%), in a ratio (v/v) 1: 999 µL. After, the activation was promoted individually with 20 mL of distilled water (24 °C ± 1°C). A subsequent mixing action was imposed on the gametes for 60 s, followed by transfer of oocytes and spermatozoa to small circular sieves. The eggs were incubated in small circular sieves with nylon nets in plastic tanks (500 L) in a recirculating system. The experimental parameters were water temperature of 24 ± 0.5 ºC, pH 7.2 ± 0.1, and hardness 50.7 ± 6.7. Fertilization rates were evaluated after the embryonic blastopore closed, approximately 12 h after fertilization (Pereira et al., 2006). The analysis was performed by counting the number of fertilized and non-fertilized oocytes; 300 oocytes from each sieve were analyzed using a binocular stereomicroscope (Q7740SZ-T, Quimis, Brazil) at 10 × magnification and a manual counter. The result was given by the formula: fertilization (%) = (number of fertilized/total oocytes) × 100. Hatching rates were observed 48 h after fertilization using a binocular stereomicroscope (10 ×) and a manual counter. The results are given by the formula hatching (%) = (number of larvae/total oocytes) × 100. The complete procedure, from anesthesia for semen collection to the acquisition of hatched larvae, is illustrated in Figure 3.

### Blood analysis

#### Blood collection

Animal blood was collected by inserting a needle (25 × 0.7 mm, 3 mL syringe) into the ventral region, caudal to the genital region, at an angle of 45 to 90º towards the ventral region of the spinal cord, to allow puncture of the caudal vein. In the control group, the blood collection time was determined according to the average anesthesia time of the fish in the MS-222 and EUG groups. A maximum of 1.5 mL was collected from each animal (*n* = 24; eight specimens per treatment).

#### Quantitation of cortisol concentrations

Blood samples were transferred to microtubes serum gel with clot activator (Microvette® 500 μL, Sarstedt, Deutschland) for plasma separation. The cortisol concentration was determined using an enzyme-linked immunosorbent assay (ELISA) according to the manufacturer's instructions (EIA DBC Kit, Canada). The methodology described by Cericato et al. (2009) was used for this analysis.

The cortisol assay precision was assessed by calculating the intra-assay coefficient of variation (CV) of 24 repeated assays. Reproducibility was evaluated by assaying the same samples on different plates and calculating the inter-assay CV. A strong positive correlation (*R^2^* = 0.9973) was observed between the evaluated values in the linear regression test. The inter- and intra-assay coefficients of variation ranged from 2 to 5% and 1–4%, respectively.

#### Leukocyte differential, glucose concentration and hematocrit

The evaluation of immune cells was performed by differential leukocyte count (Ranzani-Paiva et al., 2013). For observation, a smear was made using 0.15 µL of the blood sample from each animal. To quantify blood glucose, a 15 µL aliquot of the collected blood was analyzed in an automatic glucometer device (On Call Plus II, ACON Biotech, China).

To quantify erythrocyte volume, a microhematocrit tube (75 mm) from each sample was filled with blood to approximately 75% of its capacity. One end was sealed with fire. Tubes were centrifuged at 11200 g (g force) for 5 min. The height of the erythrocyte column was measured as a percentage of the whole blood column on a special card for reading the hematocrit.

### DNA integrity

The alkaline comet assay was performed according to Collins (2015) and Singh et al. (1988), with adaptations by Rosa-Silva et al. (2020). Blood or semen samples were homogenized in 0.7% low-melting-point agarose solution, placed on a slide pre-coated with 1.5% agarose, and placed in a lysis solution. After lysis, the slides were placed in a horizontal flask with alkaline buffer. After 20 min, DNA was developed by electrophoresis (25 V and 300 mA) for 15 min. The slides were then neutralized, fixed, and stained with silver nitrate. Analysis was performed using conventional optical microscopy and applying the analytical criteria described in the literature. To calculate the damage index (ID), the nucleoids were classified according to tail size in relation to the comet head (no damage = 0 to maximum damage = 4). The damage index (ID) of each group ranged from 0 (no damage ¼ 100 cells completely × 0) to 400 (maximum damage: 100 cells × 4). Data were expressed as percentages of the DNA damage index.

### Markers of oxidative stress - Damage markers of oxidative stress - Damage to biomolecules

To measure reduced thiol (-SH) levels in blood and semen cell fractions, we performed an Ellman reagent-based assay (Ellman, 1959). The total thiolic and non-proteic thiolic contents were measured by reacting with 10 mM 5.5-dithio-bis-(2-benzoic acid) (DTNA). For the non-protein groups, samples were mixed with trichloroacetic acid (TCA) at a final concentration of 20% and centrifuged at 10,000 *g* for 10 min to precipitate the proteins, after which the supernatant was collected. In brief, 100 μg protein/sample was added to the spectrophotometry (SpectraMax I3) plate and combined with boric acid and DTNB, spectrophotometry was then read at time point 0 using 412 nm wavelength. After 1h of incubation at room temperature (25º C), absorbance was measured again at 412 nm. First reading was deduced from the last reading, and the results were normalized to be expressed as µmol –SH mg^-1^ protein.

Carbonyl groups were determined as an index of oxidative damage to proteins as previously described by Levine et al. (1990). This method is based on the reaction of dinitrophenylhydrazine with carbonyl groups of proteins. Briefly, two tubes were prepared: a blank and DNPH tubes. In each tube, 1 mg of protein from each sample was precipitated using TCA at a final concentration of 20%. The supernatant was discarded and the pellets were resuspended in 0.2 NaOH. Blank tubes were incubated with 2M HCL, and DNPH tubes were incubated with 10 mM DNPH for 1h. The samples were precipitated again using TCA at a final concentration of 20%. The supernatant was discarded, and the pellets were washed three times using 1 ml of ethanol and ethyl acetate (1:1) and resuspended in 1mL of 8 M urea (pH 2.3). An aliquot of 200 μL was transferred to a spectrophotometry plate, and samples were read at 370 nm wavelength. Blank sample values were deduced from DNPH samples, and results are expressed as µmol Carbonyl mg^-1^ protein.

Lipid Peroxidation was determined by quantifying the reactive species of thiobarbituric acid generated by the reaction of thiobarbituric acid with lipoperoxides present in the heated acidic medium, as described by Esterbauer and Cheeseman (1990). In brief, 1 mg of proteins from samples were precipitated using TCA as 20% final concentration, supernatant was collected, and 100μL transferred to a spectrophotometry plate. An aliquot of 100μl of TBA 0.67% was added and the plate was heated to 100 ºC for 20 min using a dry block. The absorbance of the plates was read at 512 nm wavelength. A standard curve of 1,1,3-tetramethoxypropane (a lipoperoxide standard) was used for quantification. The results are expressed as nmol TBARS mg^-1^ protein.

### Statistical analysis

The normality of data and homogeneity of variances were evaluated using the Shapiro-Wilk, Kolmogorov-Smirnov, or D’Agostino-Pearson test and the Levene test, respectively. The data were transformed (LOG) when necessary, and outliers were excluded. After verifying compliance with the statistical assumptions, the data were analyzed using one-way ANOVA, and Tukey’s test was applied when a difference was observed (p<0.05). A two-way analysis of variance (Two-Way ANOVA) was used for leukocyte count, considering the effects of anesthetics (treatments), cell type, and the interaction between factors. Analyses of induction time to anesthesia and recovery from anesthesia were compared using Student’s t-test. The results of the parametric analyses are presented as bar graphs using the mean ± standard deviation. Data that were not normally distributed and/or homogeneity of variances were analyzed using the Kruskal-Wallis test followed by Dunn's test, with median differences considered (p< 0.05). The data analyzed using non-parametric analysis are presented in boxes and whisker charts (maximum and minimum). The graphs and analyses were performed using Statistical Analysis System 9.4 and GraphPad Prism 7.0 software.

## Results

**Figure 4:**
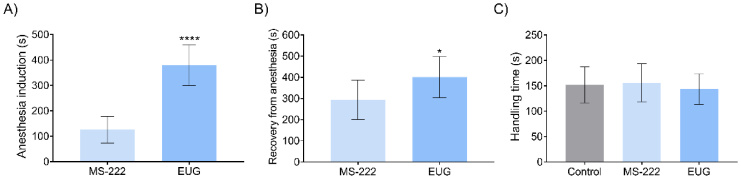
Anesthesia time of procedures in *R. quelen* anesthetized by MS-222 (n = 8), EUG (n = 8) or non-anesthetized (Control) (n = 8). A) Induction time (s) (p<0.0001); B) Recovery time (s) (p=0.0430; C) Handling time (s) (p=0.7627). Results are shown as mean ± SD. The T-Student’s test was utilized to analyze the induction time and the recovery time (*p<0.05; **p<0.01; ***p<0.001; ****p<0.0001). EUG = Eugenol.

The animals anesthetized with MS-222 achieve deep anesthesia stage faster than animals anesthetized with Eugenol (EUG) (125.4 s and 379.1 s, respectively). In contrast, the animals anesthetized with EUG required a longer period to recover from anesthesia (400.9 s) than the fish anesthetized with MS-222 (294 s, Figure 4B). The time required for semen collection was the same for all groups (Figure 4C).

**Figure 5:**
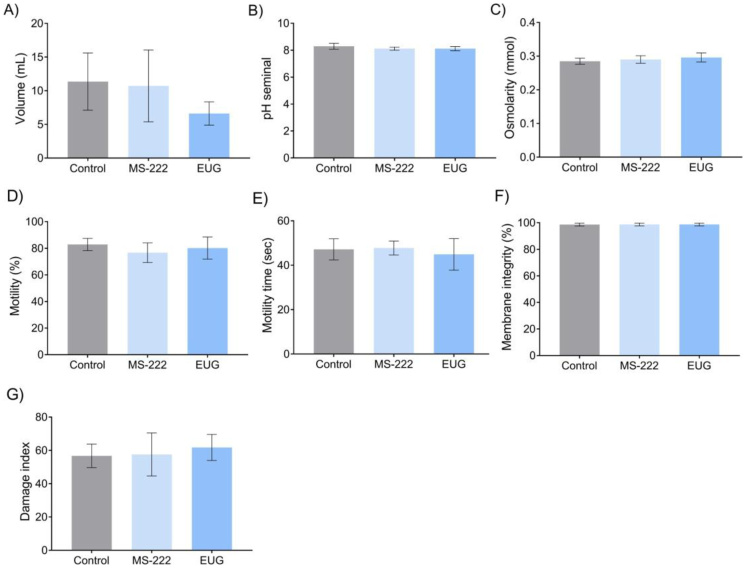
Semen parameters of *R. quelen* anesthetized by MS-222, EUG and non-anesthetized. A) volume (p=0.0644); B) pH (p=0.0831); C) osmolality (p=0.2253); D) Motility (p=0.2848); E) Motility time (p=0.6185); F) Membrane integrity (p=0.9924); F) DNA damage index (p=0.5224). Different letters above the bars indicate differences (p<0.05) in the Tukey test. EUG = Eugenol.

There were no differences in semen volume, pH, or osmolality between the groups (Figure 5). Spermatozoa morphology (Table 2) and analyses of motility, motility duration, membrane integrity, and DNA damage indices did not demonstrate any differences between the groups (Figure 5).

**Table 2:** Spermatozoa morphology of *R. quelen* anesthetized by MS-222, EUG and non-anesthetized.

**Variables (%)**	**Experimental groups**			**p-value**
	**Control**	**MS-222**	**EUG**	
Normal spermatozoa	90.06±4.88	85.33±7.63	86.81±4.07	0.3284*
Distally curled tail	0.17±0.18	0.38±0.49	0.19±0.24	0.6958**
Strongly curled tail	0.28±0.33	0.29±0.36	0.19±0.34	0.7008**
Broken tail	1.50±1.21	2.24±1.48	2.33±1.48	0.5114*
Folded tail	1.50±0.86	2.14±1.09	1.63±1.01	0.4654*
Short tail	0.61±0.65	0.81±0.81	0.44±0.65	0.7166**
Loose head	1.00±0.87	1.38±1.04	1.56±1.25	0.7166**
Macrocephaly	0.33±0.37	0.19±0.38	0.44±0.44	0.4261**
Microcephaly	0.06±0.14	0.33±0.38	0.30±0.39	0.3068**
Degenerated head	0.53±0.51	0.90±0.69	1.44±0.91	0.6486*
Proximal gout	2.17±1.53	2.67±2.33	2.11±1.13	0.2391*
Distal gout	1.89±2.41	3.33±3.53	2.56±1.95	0.8305**

EUG = Eugenol. *ANOVA; **Kruskall-Wallis.

**Figure 6:**
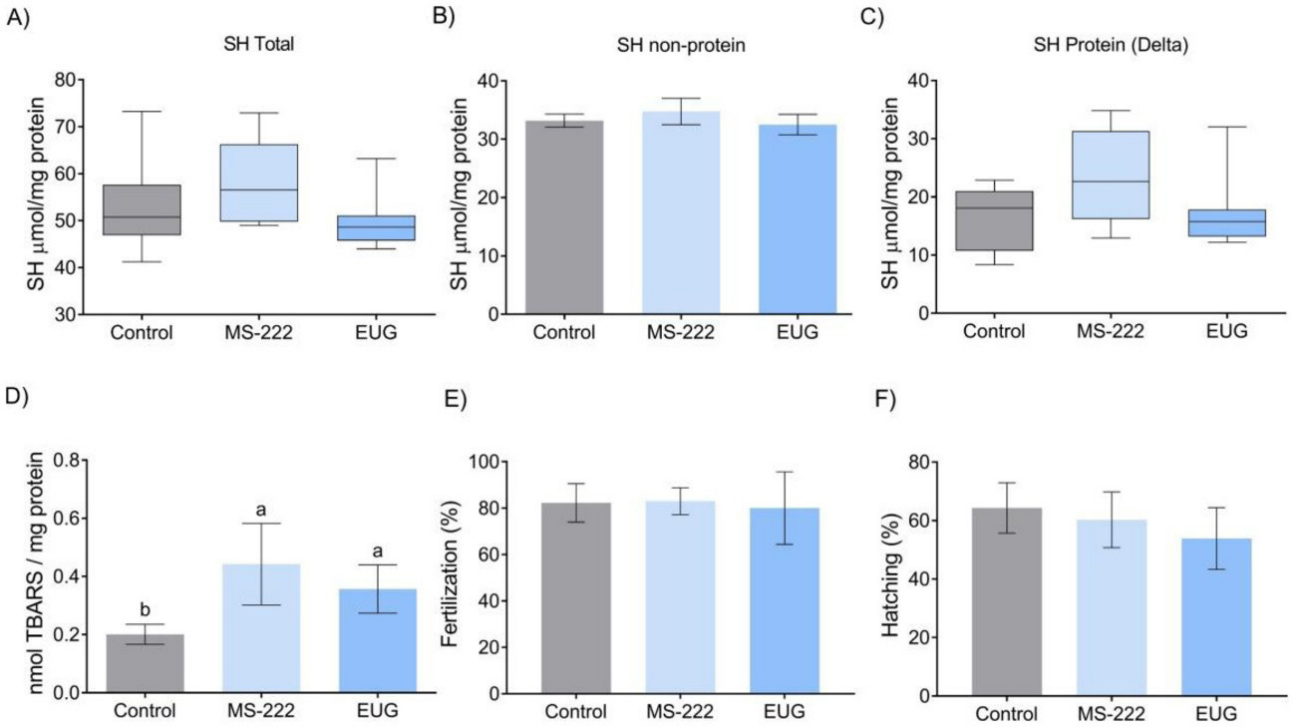
Semen antioxidant and reproduction parameters in *R. quelen* anesthetized by MS-222, EUG and non-anesthetized. A) SH total (p=0.1635), B) SH non-protein (p=0.0875), C) SH protein (p=0.2358), D) TBARS (p=0.0004); E) Fertilization rate (p=0.1507); F) Hatching rate (p=0.8765). Different letters above the bars indicate differences (*p*<0.05) in the Tukey test or Kruskal-Wallis. EUG = Eugenol

There was no difference among the groups for total reduced thiols (SH), as in the protein and non-protein fractions of semen (Figure 6AC). There was a greater concentration of TBARS in anesthetized fish than in non-anesthetized fish (Figure 6D). Fertilization, and hatching rates (Figure 6E, F) showed no differences between the groups.

**Figure 7:**
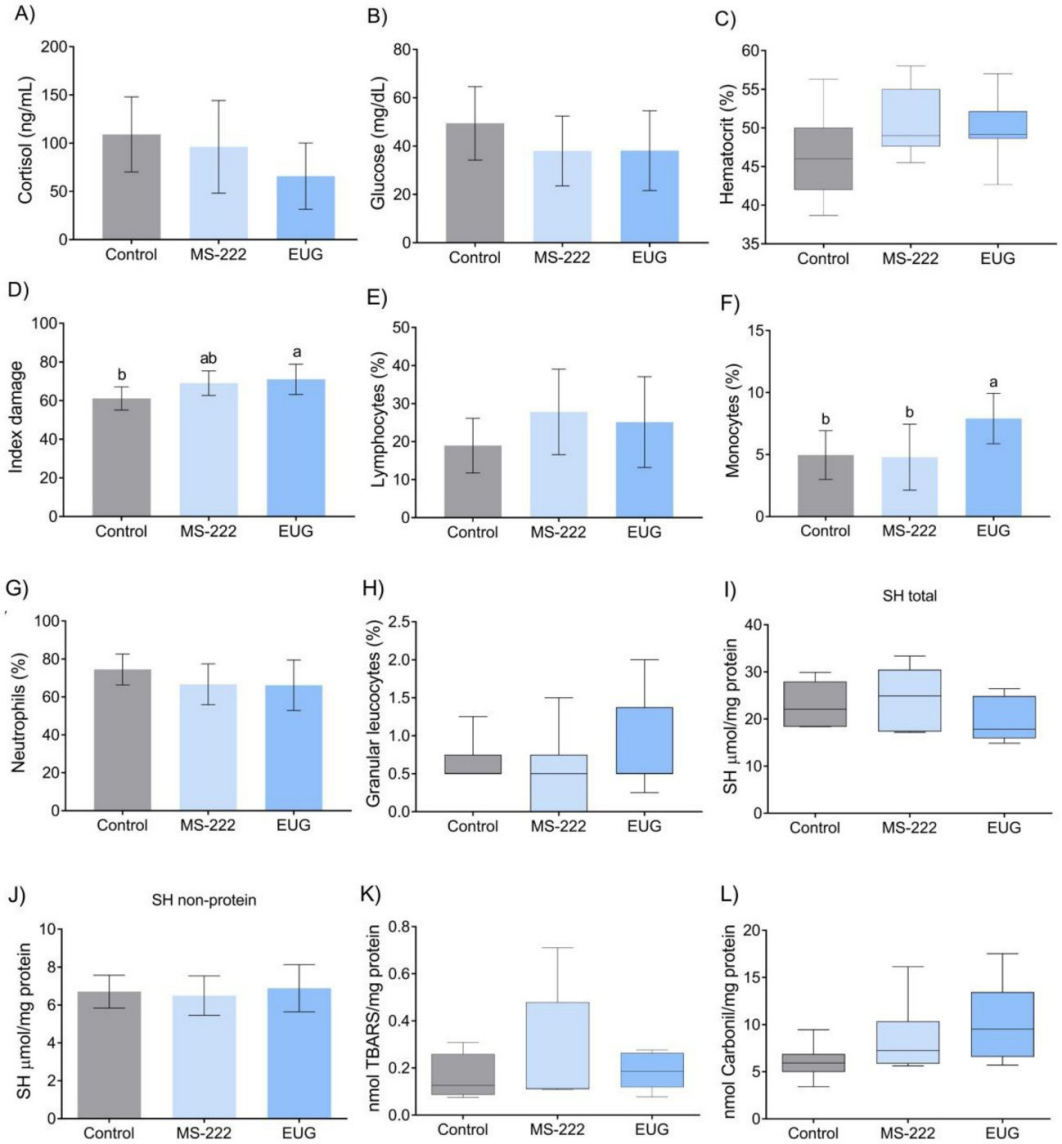
Blood parameters of anesthesia in *R. quelen* anesthetized by MS-222, EUG and non-anesthetized. A) Cortisol (p=0.1101); B) Glucose (p=0.2935); C) Hematocrit (p=0.3151); D) DNA damage index (p=0.0198); E) Lymphocytes (p=0.1101); F) Monocytes (p=0.2935); G) Neutrophils (p=0.3151); H) Granular leucocytes (p=0.0198); I) SH total (p=0.1665), J) SH non-protein (p=0.7844), K) TBARS (p=0.8169), L) Carbonil (p=0.0797). Different letters above the bars indicate differences (p<0.05) in the Tukey test or Kruskal Wallis. EUG = Eugenol.

Specimens in the EUG group exhibited a higher number of cells with DNA damage compared to the control group (Figure 7D). Plasma cortisol concentrations showed no significant differences among experimental groups (Figure 7A). Although numerical variation was observed, with slightly higher mean values in the control group and lower values in the EUG group, cortisol values were comparable among all groups, as indicated by the dispersion of individual values and error bars. Likewise, no significant differences were detected in plasma glucose concentrations or hematocrit values among groups (Figure 7B, C), with all parameters remaining within similar ranges across treatments. The analysis of blood antioxidant parameters also revealed no significant differences among groups in total reduced thiol content, non-protein thiol fractions, TBARS levels, or protein carbonyl content (Figure 7IK). In addition, the percentage of PAS-positive lymphocytes, neutrophils, and granular leukocytes also remained consistent across all groups (Figure 7E, G, H). However, fish anesthetized with EUG showed a significantly higher percentage of monocytes compared to those anesthetized with MS-222 and the control group (Figure 7F).

## Discussion

The use of anesthesia in fish reproductive management is widely recognized for enhancing both animal welfare and operator safety. In this study, MS-222 and eugenol did not compromise key reproductive parameters such as fertilization and hatching rates in *Rhamdia quelen*, supporting their functional suitability in reproductive protocols.

Oxidative stress represents one of the main damages suffered by fish spermatozoa (Cabrita et al., 2014; Sandoval‐Vargas et al., 2020; Souza et al., 2024). Several factors can favor this condition, such as exposure to pollutants and xenobiotics, including anesthetics (Velisek et al., 2011; Guptha et al., 2016; Readman et al., 2017; Li et al., 2025). Although enzymatic activities are reliable indicators of oxidative stress, each organ, tissue, and organism may respond differently under pro-oxidant conditions by gathering enzymatic and non-enzymatic responses against the generation of reactive oxygen species (ROS) generated (Amado et al., 2009). SH group content is an important marker of protein peroxidation. The decrease in their content over time may reflect protein damage, whereas the increase may indicate the intensification of antioxidative defenses against free radicals. In this study, we did not observe a difference in the content of SH groups in the spermatozoa of anesthetized animals. However, when used with anesthetics, the semen showed increased lipid peroxidation levels. Other studies have reported similar results. Anesthesia with MS-222 causes lipid peroxidation and a decrease in several antioxidant enzymes in rainbow trout tissues (Velisek et al., 2011). In spotted knifejaw (*Oplegnathus punctatus*), clove oil induced hepatic oxidative stress and improved the antioxidant defense capacity to maintain homeostasis during anesthesia and recovery (Jia et al., 2022). In this context, oxidative stress analysis is essential, particularly considering a recent zebrafish study demonstrating that chronic etomidate exposure induces pronounced oxidative stress, disrupts reproductive tissues, and activates the mTORC1 pathway, leading to PCOS-like (polycystic ovary syndrome) ovarian alterations and transgenerational adverse effects (Li et al., 2025).

Anesthesia in fish should be rapidly induced, with the appropriate depth reached within 3 minutes to minimize stress and prevent hyperactivity. Recovery should occur within 5 minutes after transferring to clean water, with 10 minutes considered the maximum acceptable duration (Ross and Ross, 2008). Based on these criteria, the MS-222 concentration used in the present study was deemed effective for inducing and recovering from deep anesthesia. In contrast, eugenol required a longer period to induce loss of equilibrium, reach stage IV anesthesia, and achieve full recovery compared to MS-222. However, its recovery time remained under 10 minutes, in line with observations in *Oncorhynchus mykiss* (Wagner et al., 2002) and the spotted sea bass, *Lateolabrax maculatus* (He et al., 2020). In zebrafish, however, eugenol induces anesthesia more quickly than MS-222, but results in longer and progressively increasing recovery times with repeated exposure (Ayala-Soldado et al., 2024). Also, during *Lisa ramada* female’s anesthesia, clove oil produced faster anesthesia, and the time required for fish recovery was longer in comparison with MS-222-exposed fish (Ayyat et al., 2025). Various biological and environmental factors can influence the effectiveness of anesthesia in fish (Ross and Ross, 2008), including water parameters (Gomes et al., 2011). Such discrepancies may also reflect species-specific physiological responses to anesthetics, including differences in metabolic rate, sensitivity, and tolerance (Minaz et al., 2025). According to Félix et al. (2023), there is a negative linear relationship between water temperature and both anesthesia induction and recovery times when using monoterpene-based anesthetics, indicating that higher temperatures accelerate both processes. In the case of MS-222, water temperature shows minimal effect on the optimal dose and minimum effective concentration in Asian seabass, but it significantly affected pharmacokinetics, particularly by increasing the rate of drug elimination (Hsu et al., 2023). This is likely due to the temperature-dependent acceleration of physiological functions such as absorption, distribution, and clearance. Therefore, temperature should be considered a critical confounding factor when evaluating anesthetic protocols in fish (Félix et al., 2023).

The stress response is triggered immediately upon the perception of a stressor. The neuroendocrine system's initial reaction involves the release of catecholamines, followed by corticosteroids, primarily cortisol, which is considered the most important stress indicator in fish (Gorissen and Flik, 2016; Bagheri et al., 2025). In *Rhamdia quelen* males, the basal cortisol concentration is 15.86 ng/mL; however, under acute stress, levels can rise sharply, reaching 158.12 ng/mL in males and 207.0 ng/mL in females within one hour of handling (Barcellos et al., 2001). Although MS-222 and EUG reduced cortisol concentrations in the present study, they did not differ significantly from those in the control group. As a complementary indicator of stress, hematocrit and glucose are widely used (Sopinka et al., 2016). Rapid increases in plasma glucose levels are mediated by the release of catecholamines, which are readily available for use by the skeletal muscles (Schreck and Tort, 2016; Hsu et al., 2023). In the present study, glucose and cortisol levels followed a similar pattern across treatments, with no significant differences among groups, indicating that this physiological association was maintained at basal levels. A comparable relationship was reported by Junmahasathien et al. (2025), in which plasma glucose levels increased in parallel with cortisol elevations when *Ocimum basilicum* and MS-222 were tested in *Carassius auratus*.

An increased hematocrit percentage following anesthetic administration has been most commonly reported in *Seriola dumerilii* (Maricchiolo and Genovese, 2011), *Rhamdia quelen* (Gressler et al., 2014), and *Lophiosilurus alexandri* (Boaventura et al., 2020). Elevated hematocrit levels may result from hypoxia and/or the release of red blood cells by the spleen in response to acute stress mediated by catecholamines (Tort et al., 2002). However, the hematocrit values observed in the present study were similar across all groups. Zahran et al. (2021) reported similar findings in *Oreochromis niloticus* anesthetized with eugenol at 30 mg L^-1^. Our results may reflect the potential stress-inducing effects of anesthetic agents. Alternatively, the lack of significant differences could be attributed to the elevated basal cortisol levels observed in all groups, leading to comparable glucose and hematocrit levels.

The alkaline comet assay is widely used to evaluate DNA damage in genotoxicity tests and DNA damage and repair mechanisms. This assay enables alkaline treatment and electrophoresis to detect single- or double-stranded breaks in DNA, which are alkali-labile sites, through the alkylation of electronegative DNA groups and cross-links (Collins, 2015). Studies have demonstrated the ability of eugenol to protect against DNA strand breaks in mammals (Yogalakshmi et al., 2010) and humans (Salah et al., 2019). In the present study, the comet assay performed on the eugenol group blood showed higher numbers of erythrocytes with DNA damage than in non-anesthetized animals. Although eugenol is often used in aquaculture, to our knowledge, there are few studies on its cytotoxicity and genotoxicity in fish. A study on *Oreochromis niloticus* and *Astyanax lacustres* showed that anesthesia with eugenol at 150 mg L^-1^ caused genotoxicity in both species (Nascimento et al., 2020). The mechanisms involved have not yet been fully elucidated; however, eugenol-induced genotoxicity may be partially related to oxidative damage. Eugenol undergoes biotransformation into electrophilic quinone methides, potentially resulting in oxidative base damage and formation of DNA adducts (Martins et al., 2018).

Acute, short-term stress can enhance immune function as an adaptive psychophysiological mechanism, increasing host protection against injury or infection. This transient response is marked by cortisol-driven neutrophil mobilization, extended neutrophil survival, and the upregulation of chemokines and cell recruitment pathways, collectively promoting systemic immune preparedness without compromising long-term homeostasis (Klak et al., 2024). It is crucial to determine whether a given stressor enhances or suppresses immune function, as the outcome of the immune response dictates whether stress–immune interactions will have beneficial or detrimental effects on health (Dhabhar, 2014). According to Pietsch et al. (2025), the expression pattern of cortisol-related genes varies depending on the type of stressor (e.g., air exposure, feed reward, confinement), indicating that not all stressors elicit the same hormonal response. Stress-induced alterations in the neutrophil-to-lymphocyte ratio are a commonly used indicator of immune system disturbance (Seibel et al., 2021). Our findings align with previous studies demonstrating that acute stress commonly leads to lymphopenia and monocytopenia, while simultaneously increasing neutrophil proportions (Yada and Tort, 2016). In fish, these hematological shifts can occur rapidly, often within minutes of handling (Barton and Iwama, 1991). Pickering et al. (1982) showed that even a brief, 2-minute handling event triggered marked lymphocyte depletion within 8 hours, with immune recovery extending up to 72 hours. Similarly, Barcellos et al. (2004) reported that acute stress in *Rhamdia quelen* reduced the number of circulating lymphocytes, monocytes, and special granulocytic cells (SGCs), while promoting relative neutrophilia. Notably, Dhabhar (2014) proposed that such leukocyte redistribution may reflect an adaptive immune strategy, where cells migrate from circulation to peripheral tissues-particularly the skin and subcutaneous regions-to reinforce barrier immunity during perceived threats. In our study, monocyte counts were significantly higher in the EUG-treated group, suggesting that eugenol may attenuate the immunosuppressive effects of acute stress in *R. quelen*. This protective effect was not observed in the MS-222 or non-anesthetized groups. Despite this, all groups exposed to handling stress displayed an elevated proportion of neutrophils, reinforcing the notion that acute stress elicits a conserved, innate immune mobilization, even in the presence of anesthetic agents.

In the present study, MS-222 was associated with rapid anesthesia induction and recovery. Although its use increased spermatozoa lipid peroxidation, fertilization and hatching rates were not affected. Eugenol (EUG) exhibited genotoxic potential in *R. quelen* males, which warrants further investigation; however, no detrimental effects on fertilization or hatching efficiency were observed. Consistent with the recommendations of Minaz and Félix (2025), these findings highlight that the selection of anesthetic agents should extend beyond anesthetic performance and reproductive outcomes, incorporating the evaluation of potential sublethal physiological and cellular effects. Accordingly, the final choice of anesthetics should integrate experimental evidence with regulatory compliance, availability, cost-effectiveness, ease of use, and safety for both users and the environment.

## Conclusion

MS-222 and EUG anesthetics during semen collection caused minor variations in semen quality and stress parameters but did not affect reproductive efficiency. Thus, both anesthetics can be used for the reproductive handling of *R. quelen* males.

## Data Availability

Research data is only available upon request.
